# Viscosity of bridgmanite determined by in situ stress and strain measurements in uniaxial deformation experiments

**DOI:** 10.1126/sciadv.abm1821

**Published:** 2022-03-30

**Authors:** Noriyoshi Tsujino, Daisuke Yamazaki, Yu Nishihara, Takashi Yoshino, Yuji Higo, Yoshinori Tange

**Affiliations:** 1 Institute for Planetary Materials, Okayama University, 27 Yamada, Misasa, Tottori 682-0193, Japan.; 2 Geodynamics Research Center, Ehime University, 2-5 Bunkyo-cho, Matsuyama, Ehime 790-8577, Japan.; 3 Japan Synchrotron Radiation Research Institute, 1-1-1 Kouto, Sayo, Hyogo 679-5198, Japan.

## Abstract

To understand mantle dynamics, it is important to determine the rheological properties of bridgmanite, the dominant mineral in Earth’s mantle. Nevertheless, experimental data on the viscosity of bridgmanite are quite limited due to experimental difficulties. Here, we report viscosity and deformation mechanism maps of bridgmanite at the uppermost lower mantle conditions obtained through in situ stress-strain measurements of bridgmanite using deformation apparatuses with the Kawai-type cell. Bridgmanite would be the hardest among mantle constituent minerals even under nominally dry conditions in the dislocation creep region, consistent with the observation that the lower mantle is the hardest layer. Deformation mechanism maps of bridgmanite indicate that grain size of bridgmanite and stress conditions at top of the lower mantle would be several millimeters and ~10^5^ Pa to realize viscosity of 10^21–22^ Pa·s, respectively. This grain size of bridgmanite suggests that the main part of the lower mantle is isolated from the convecting mantle as primordial reservoirs.

## Teaser

Direct viscosity measurements of bridgmanite demonstrate viscosity contrast in Earth’s mantle and deep mantle evolution.

## INTRODUCTION

Rheological properties, including the viscosity of the constituent minerals, are fundamental to comprehending the deformation and the dynamics of Earth mantle. The viscosity-depth models of Earth’s mantle proposed from many geophysical observations (*1*–*4*) indicate that the lower mantle has the largest viscosity among all the mantle layers. Viscosities of the transition zone and that of the top of the lower mantle have been assessed 10^19–21^ and 10^21–22^ Pa·s, respectively, generating a viscosity contrast of one to two orders of magnitude. To interpret the observed viscosity profiles of Earth’s mantle in terms of mineral physics, it is indispensable to constrain the rheological properties of the constituent minerals, especially those of bridgmanite, which is the most dominant mineral in the lower mantle.

In Earth’s mantle, the dominant deformation mechanisms are generally considered to be diffusion creep and/or dislocation creep. The total strain (ε_total_) during deformation is the sum of the strains of diffusion creep (ε_dif_) and dislocation creep (ε_dis_), and viscosity (η) is defined as $\eta =\sigma /{\overset{\cdot }{\varepsilon }}_{\text{total}}$ where σ and ${\overset{\cdot }{\varepsilon }}_{\text{total}}$ are stress and total strain rate, respectively. Diffusion creep of bridgmanite has been studied mainly by diffusion experiments (*5*–*7*) under deep mantle conditions, whereas dislocation creep of bridgmanite is still unclear. Recent measurements of dislocation recovery rates of ringwoodite and bridgmanite under both dry and wet conditions by annealing experiments (*8*) show that the nearly water-saturated conditions with water content of ringwoodite of 1 to 2 weight % (wt %) are required to explain the observed viscosity contrast between the transition zone and the lower mantle. On the other hand, the average water content of the transition zone is estimated to be less than 0.2 wt % from comparison with electrical conductivity measurements (*9*, *10*) and geoelectromagnetic induction studies (*11*). Therefore, there are large uncertainties with previous viscosity evaluations using the indirect method of dislocation recovery measurements (*8*), and it is essential to quantitatively investigate the flow laws of mantle minerals in the dislocation creep regime based on the direct measurements.

In situ stress measurements and texture development measurements in the diamond anvil cell with presynthesized bridgmanite aggregate without annealing (*12*) and sample synthesized in situ from enstatite, olivine, and ringwoodite with annealing (*13*) were conducted at only room temperature during compression. In situ stress relaxation experiments of bridgmanite aggregate using a DIA apparatus (*14*) were performed up to 1027 K and reported flow laws of dislocation glide. In situ large shear deformation experiments of bridgmanite and ferropericlase two-phase system at high pressure and high temperature using the rotational Drickamer apparatus (RDA) (*15*) were conducted. To estimate flow law of pure climb creep of bridgmanite, in which strain was produced by dislocation climb motion, dislocation dynamics (DD) simulations (*16*) were conducted. However, flow laws of bridgmanite in dislocation creep regime by the direct measurements are still not determined.

To determine the viscosity of bridgmanite in the dislocation creep region, we carried out in situ stress and strain measurements of MgSiO_3_ bridgmanite during uniaxial deformation at temperatures of 1473 to 1673 K and pressures of 23 to 27 GPa using the Kawai-type cell assembly with both the deformation DIA–type apparatus (D-DIA), hereafter we called as “KATD” (*17*, *18*), and the deformation-111 (D111) apparatus (*19*, *20*) at the synchrotron x-ray radiation facility (Materials and Methods).

## RESULTS

**Fig. 1.**
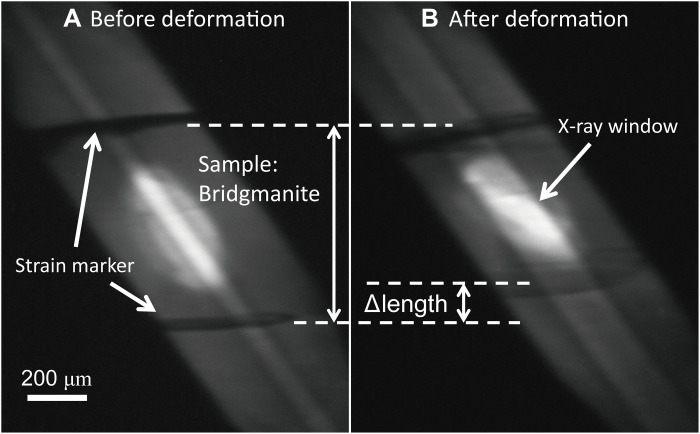
X-ray radiographs of bridgmanite deformation experiments in run KM19 in the D111 apparatus. (**A**) Before deformation. (**B**) After deformation. Total strain reached 21.0%. Delta length is the shortened length of the sample by uniaxial deformation.

**Fig. 2.**
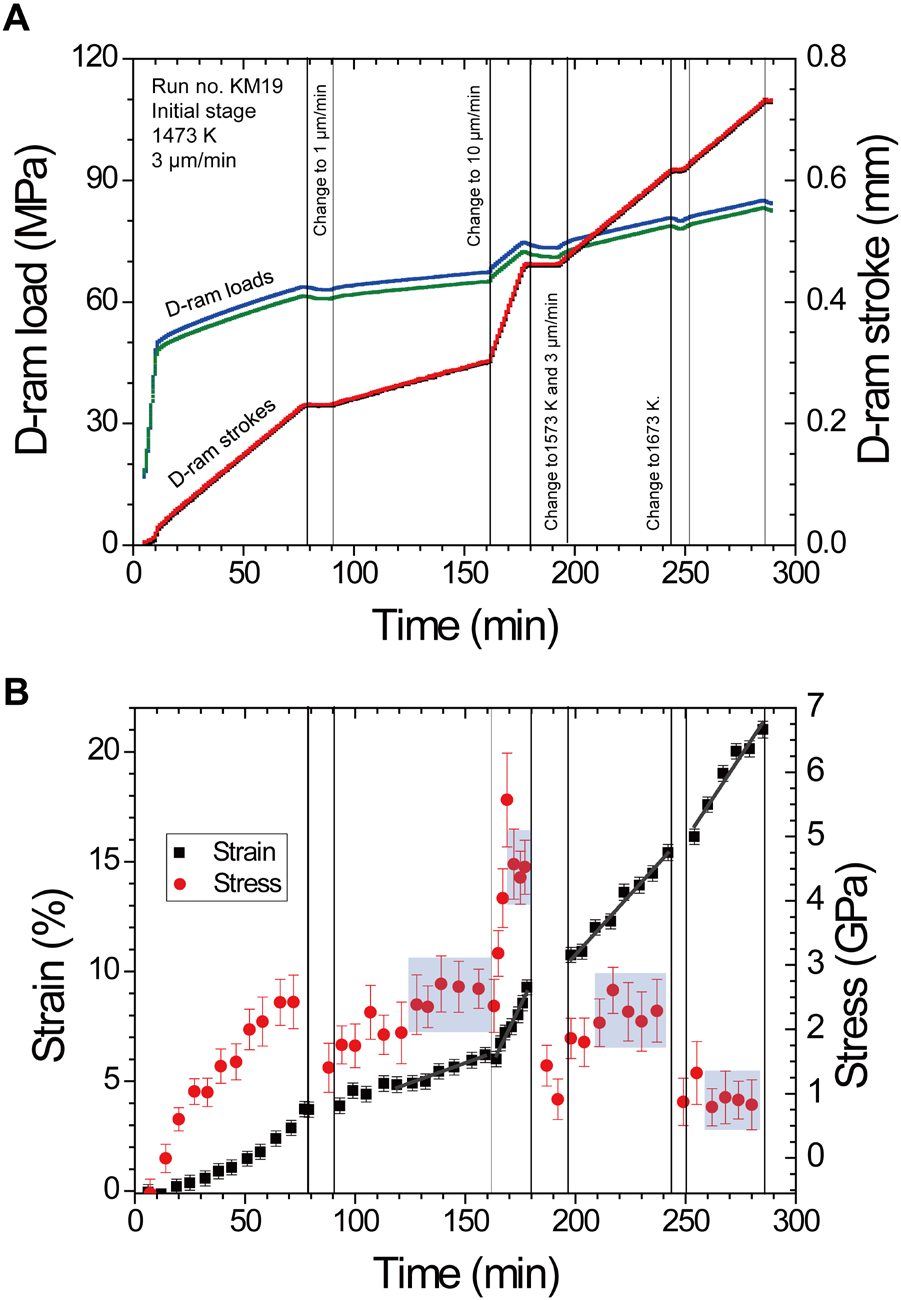
Time evolution of the differential ram and stress and strain of sample in run KM19. (**A**) The load and stroke of (D-ram) against time. (**B**) Stress and strain of sample with time. Time “0” is the onset of movement of differential ram. In the first stage from 0 to 80 min, deformation is conducted at differential ram speed of 3 μm/min at 1473 K. From 90 to 160 min and from 160 to 180 min, the second and the third stages of deformation were continuously performed at speeds of 1 and 10 μm/min at 1473 K, respectively. From 197 to 241 min and from 250 to 285 min, the fourth and the fifth stages of deformation were conducted at 3 μm/min and at 1573 and at 1673 K, respectively. (A) Black and red lines, which almost overlap each other, show top and bottom of differential ram strokes, respectively. Blue and green lines show top and bottom of differential ram loads, respectively. (B) Strain (black symbol) and stress (red symbol) with progressing time. Except for the first stage, steady-state creep was realized as shown by blue regions.

**Fig. 3.**
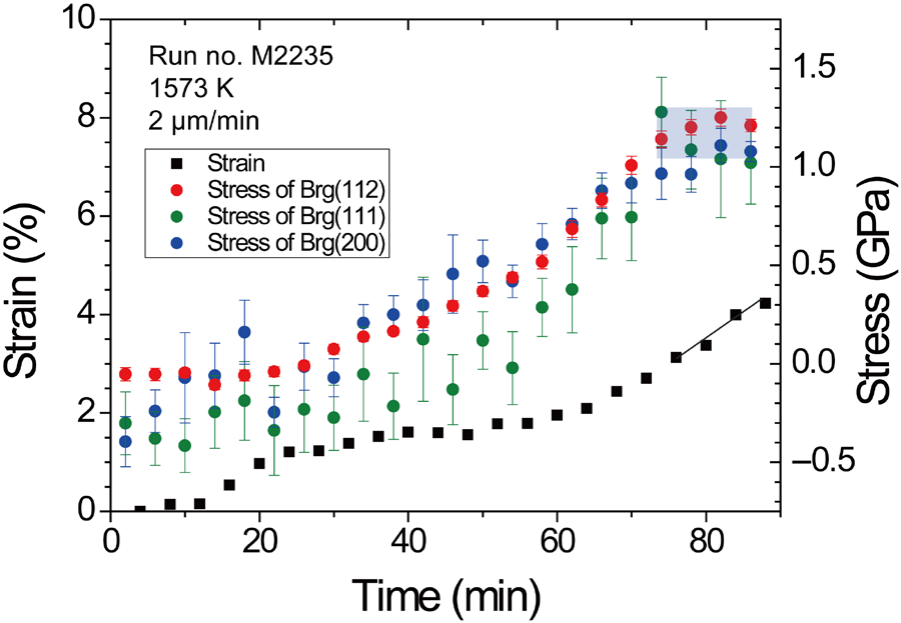
Strain and stresses of bridgmanite with progressing time by KATD apparatus, respectively. Black symbol represents strain of bridgmanite. Red, green and blue symbols denote stress from (112), (111), and (200) diffraction of bridgmanite. Steady-state creep was realized at the last stage with small strain compared with D-111 apparatus as shown by blue regions.

Stress and strain rate in the deformation experiments encompassed from 0.25 to 4.5 GPa and from 1.6 × 10^−6^ to 1.5 × 10^−4^ s^−1^, respectively (see table S1). The maximum strain of bridgmanite by using the KATD was 6.8%, whereas it reached 30.1% by using the D111 apparatus as shown in Fig. 1. Deformation in the D111 apparatus was carried out at several steps of temperature and displacement rate of differential rams to determine the stress exponent *n* and the activation enthalpy H^*^ at steady-state creep by each individual experiment as shown in Fig. 2A. As shown in Fig. 2B, except for the initial deformation stage with 3 μm/min at 1473 K, steady-state creeps were observed. In the second and third deformation stages, different displacement rates of the differential ram at the same temperature were performed to determine stress exponent *n* at constant temperature. In the latter two stages, temperature was increased at constant displacement rate of the differential ram from 1473 and 1573 K and further to 1673 K to determine activation enthalpy H^*^. The large strain obtained by the D111 apparatus enables us to realize in situ measurements of several steady-state creep conditions in each experiment as shown in Fig. 2. By the uniaxial deformation using the D111 apparatus with large strain as shown in fig. S7, the peak intensity ratio of each diffraction against azimuthal angle that related to crystallographic preferred orientation (CPO) was also observed. The (200) peak intensity increases parallel to uniaxial deformation direction, and (020) and (112) peak intensities increase perpendicular to uniaxial deformation direction. This means that dominant slip plane of bridgmanite in this study would be (100) plane, which is consistent with a previous study by shear deformation (*17*). To minimize the effect of CPO on the calculation of stress, strain was limited as bare minimum; hence, each experiment in the KATD was conducted at only one of the prescribed temperatures and differential ram displacement rates. Therefore, we estimated stress from diffraction peaks of (111), (200) of bridgmanite to evaluate the dominant slip system, and (112) peak as shown in Fig. 3. On the other hand, stress from (002) and (110), which is also useful to estimate the dominant slip system by the elastic-viscoplastic self-consistent (EVPSC) (*19*), cannot be calculated in this study because (002) + (110) doublet cannot be separated as shown in fig S5. The EVPSC models of a single dominantly active slip system with (100) slip plane up to sample strain of 20% (*19*) suggested that stress from (200) was twice larger than that from (111) in (100)[010] slip system and that the stress difference between (200) and (111) by (100)[001] slip system is smaller than that by (100)[010] slip system. In this study, obvious stress difference along (111), (112), and (200) was not observed at steady-state creep. Although error of estimated stress is large to determine the slip system by the EVPSC model, (100)[001] slip system, which is consistent with shear deformation experiments (*17*), is the most presumable candidate of the dominant slip system.

**Fig. 4.**
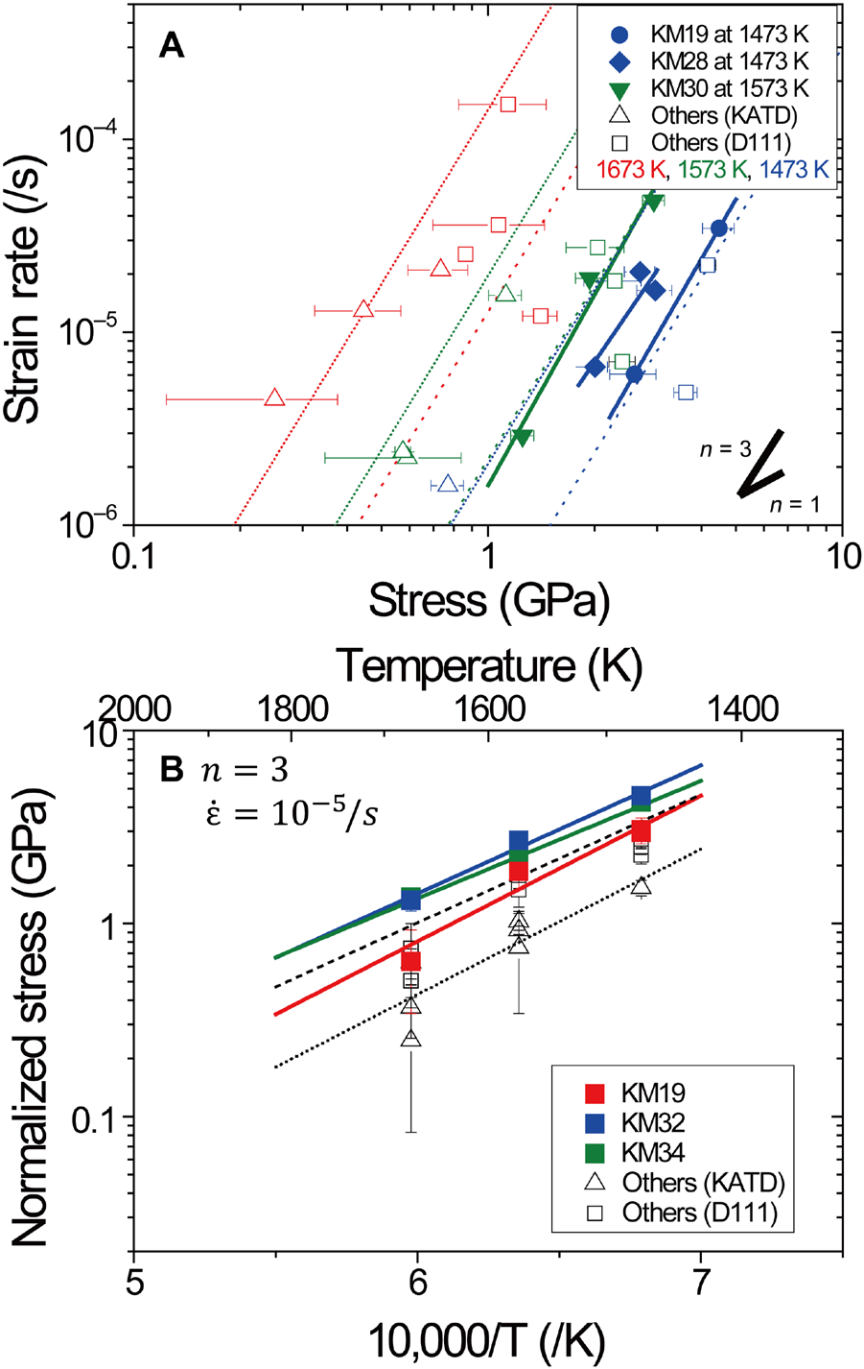
Creep strength of bridgmanite. (**A**) Relationship between stress and strain rate. (**B**) Relationship between the normalized stress and temperature. (A) Filled circles, diamonds, and inversed triangles represent stress and strain rate data at 1473 K in run KM19, at 1473 K in run KM28, and at 1573 K in run KM30, respectively, together with the best fitting lines (solid lines). Red, green, and blue colors denote data at temperatures of 1673, 1573, and 1473 K, respectively. (B) Solid lines show the best fit of data obtained in runs KM19, KM32, and KM34. Open triangles and squares represent the data from the experiments in the KATD and the D111 apparatuses with the best fitting lines (dashed and dotted lines), respectively. Note that the stress was normalized to constant strain rate of 10^−5^/s, assuming the dislocation creep of stress exponent *n* = 3.

Figure 4A shows all the data of stress and strain rate relationship of bridgmanite in the steady-state creep acquired by using both the KATD and the D111 apparatuses. The stress determined by using the KATD is slightly lower than those by using the D111 apparatus under the same temperature and strain rate conditions. This inconsistency could be caused by difference of thermal gradient through the sample produced from the difference in cell designs (Materials and Methods). Experimental data in both apparatuses show that stress at constant strain rate decreases with increasing temperature and that the strain rate at constant temperature increases with increasing stress. The flow law of dislocation creep is

$${\overset{\cdot }{\varepsilon }}_{\text{dis}}=A\sigma^{n}\text{exp}(-\frac{H^{*}}{\mathrm{RT}})$$ (1)

where *A, n*, H^*^, *R*, and *T* are the constant, the stress exponent, the activation enthalpy (kJ/mol), the gas constant (J/K/mol), and the temperature (K), respectively. By fitting to Eq. 1, stress exponents *n* are obtained to be 3.2, 2.7 ± 1.2, and 3.3 ± 0.6 by the individual experiment of runs KM19, KM28, and KM30. These values are consistent with theoretical and experimental values of 3 and ~3.5 on olivine (*21*, *22*), respectively. Flow law of pure climb creep by DD simulations (*16*) suggested strain rate of bridgmanite in pure climb regime with dislocation density of 10^12^ m^−2^ at stress of 1 GPa and 1900 K was 10^−10^ s^−1^, which is five orders of magnitude lower than the present estimation under the same condition. CPO also is developed by large strain as shown in fig. S7. The dominant deformation mechanism in this study is not consistent with pure climb creep. Therefore, it is concluded that the dominant deformation mechanism of bridgmanite in this study is dislocation creep, which means that strain is produced by dislocation glide. Figure 4B shows temperature dependency of the stress normalized to constant strain rate of 10^−5^ s^−1^ assuming *n* = 3, which derives activation enthalpies to be 456 ± 76 kJ/mol for KM19, 382 ± 46 kJ/mol for KM32, and 350 ± 35 kJ/mol for KM34 (see table S2). Fitting all the data by the D-111 apparatus and the KATD also yields 385 ± 59 and 405 ± 77 kJ/mol of H*, respectively. Activation enthalpy of 183 to 236 kJ/mol reported by in situ stress relaxation experiments in dislocation glide regime is substantially smaller than that of this study, which means that deformation mechanism of bridgmanite in this study would be different from dislocation glide (*14*). At similar temperature against melting temperature and strain rate condition of olivine, climb-controlled dislocation creep is known to be dominant (*23*). In bridgmanite, diffusion experiments (*7*) reported that the atoms with the lowest diffusion coefficient were both Si and Mg. Activation enthalpy of Si and Mg self-diffusion in bridgmanite (*5*–*7*) is reported to be 300 to 400 kJ/mol including uncertainty, which is in general agreement with the activation enthalpies obtained in this study. This indicates that the main rate-limiting process during deformation in bridgmanite is Si and/or Mg self-diffusion; hence, the dominant deformation mechanism of bridgmanite in this study is climb-controlled dislocation creep.

## DISCUSSION

### Viscosity contrast in Earth’s mantle minerals

**Fig. 5.**
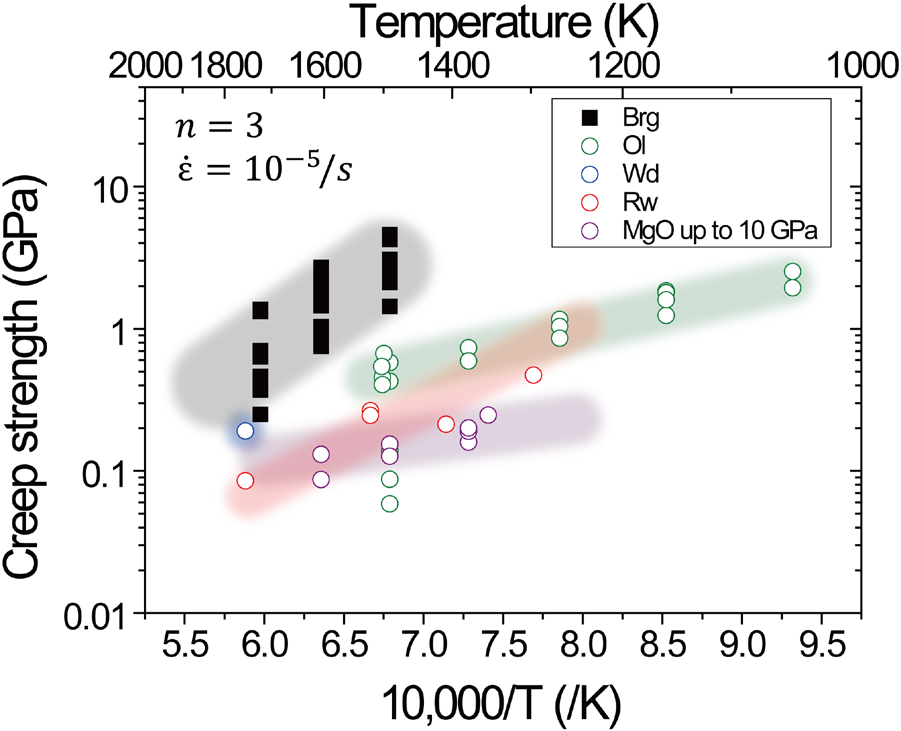
Comparison of creep strengths at strain rate of 10^−5^/s in dislocation creep with other mantle minerals reported in previous works using the D-DIA–type apparatus. Black solid squares show the strength for bridgmanite determined in this study. Open green, blue, red, and purple circles show the strength for olivine (Ol) (*24*–*26*), wadsleyite (Wd) (*27*), ringwoodite (Rw) (*28*), and MgO (*29*), respectively.

Mantle viscosity is dominated by the rheological properties of olivine and its high-pressure polymorphs bridgmanite and ferropericlase because they are the major constituents throughout the mantle. Figure 5 shows the strengths in dislocation creep of bridgmanite determined in this study by using the D111 and the KATD apparatuses and those of other mantle minerals in previous studies by using uniaxial deformation geometry in the D-DIA apparatus (*24*–*29*). Note that the creep strengths of mantle minerals determined in the RDA (*15*) are considerably higher than those in the D-DIA apparatus when stresses from same diffraction peaks of same minerals are compared because of complex deformation geometry in the RDA (see fig. S8) (*28*); therefore, it is appropriate that the creep strengths by this study are compared with that of a previous study by D-DIA because deformation geometry of this study is the same as that of the D-DIA apparatus. Creep strength data in Fig. 5 were obtained under nominally dry conditions, although ringwoodite and wadsleyite contained small amount of water (0.029 to 0.1 wt %) (*27*, *28*). The creep strength of bridgmanite is the highest among those of olivine, its high-pressure polymorphs, and periclase. This result is supported by the additional deformation experiment for the observation of the direct viscosity contrast, in which both samples of bridgmanite and ringwoodite were deformed under dry conditions in a serial arrangement along the deformation axis as shown in fig. S2B. This result also supports that bridgmanite is considerably harder than ringwoodite even under nominally dry conditions (fig. S9). Quantitatively, Fig. 5 shows that the creep strength of bridgmanite is approximately one order of magnitude larger than that of ringwoodite. Therefore, the observed viscosity variation between the mantle transition zone and top of the lower mantle would be explained by the creep strength contrast between bridgmanite and ringwoodite even under nominally dry conditions, which is consistent with estimates based on their electrical conductivities (*9*–*11*).

### Deformation mechanism maps of bridgmanite

**Fig. 6.**
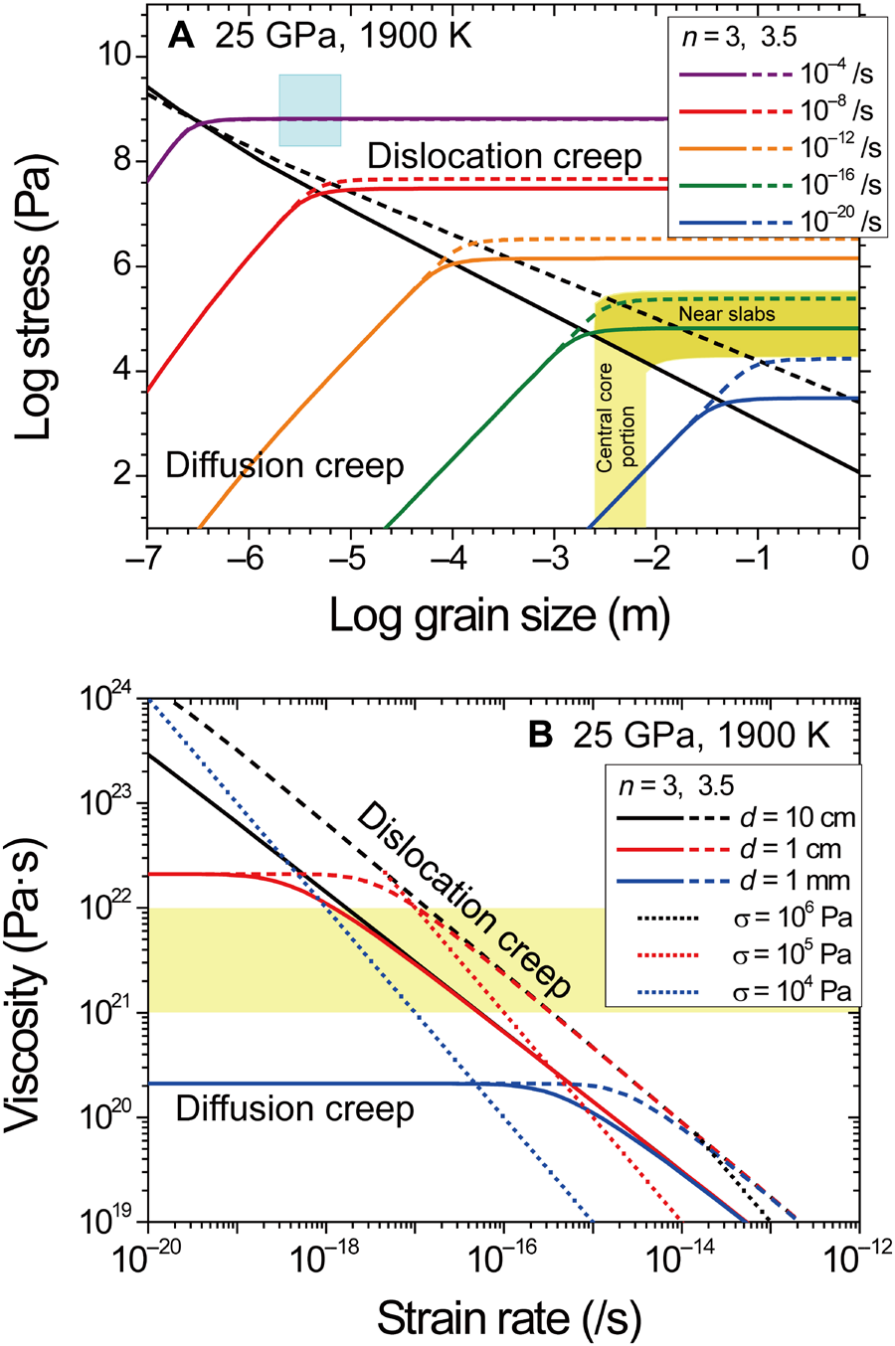
Deformation mechanism maps for bridgmanite at 25 GPa and 1900 K constructed by using the parameters determined in the present study for dislocation creep (table S2) and the previous diffusion study for diffusion creep (*5*). (**A**) Stress and grain size relationship with constant strain rates. Black solid and dashed lines show boundaries between dominant deformation mechanisms of the dislocation creep and the diffusion creep regimes with stress exponents *n* of 3 and 3.5, respectively. Light blue, yellow, and dark yellow regions denote the experimental conditions and the region dominated by diffusion creep and dislocation creep with observed viscosity range of 10^21–22^ Pa·s, respectively. (**B**) Strain rate and viscosity relationship with constant grain size (solid and broken lines) and constant stress (dotted lines).

We constructed deformation mechanism maps of bridgmanite at 1900 K and 25 GPa as shown in Fig. 6 based on the present results for dislocation creep and previous results for diffusion creep as shown in the following equation (*23*)

$${\overset{\cdot }{\varepsilon }}_{\text{dif}}=\frac{42\sigma \Omega }{\mathrm{RT}d^{2}}(D_{l}+\frac{\pi }{d}\delta D_{\text{gb}})$$ (2)

where Ω is the atomic volume (m^3^/mol), *d* is the grain size (m), *D*_l_ is the lattice diffusion coefficient (m^2^/s), and δ*D*_gb_ is the grain boundary diffusion coefficient (m^3^/s). Lattice diffusion coefficient and grain boundary diffusion coefficient by previous annealing experiments (*5*) (see table S2) were used to calculate viscosity of bridgmanite in diffusion creep regime. Temperature dependence on strain rate is mainly derived from those of lattice and grain boundary diffusion coefficients, which are the most important parameters in diffusion creep regime. To realize viscosity of 10^21–22^ Pa·s for the top of the lower mantle, stress magnitude and strain rate are required to be 2 × 10^4^ to 3 × 10^5^ Pa and 2 × 10^−18^ to 3 × 10^−16^ s^−1^ in the grain size–insensitive dislocation creep regime, respectively, whereas a grain size of 3 to 8 mm is demanded in the stress-independent diffusion creep regime as shown in Fig. 6. These stress magnitudes of bridgmanite are consistent with those in the upper mantle estimated from flow laws of olivine (*30*, *31*). Assuming the plate motion of 1 cm/year and Earth’s mantle thickness of ~3000 km, the average strain rate of the mantle is calculated to be approximately 10^−16^ s^−1^, in accord with the required strain rate in dislocation creep of bridgmanite. The observed viscosity and the expected strain rate at the top of lower mantle can be reasonably explained by using the deformation mechanism maps of bridgmanite; therefore, it is concluded that rheology of the lower mantle is dominated by bridgmanite, which means that bridgmanite forms the load-bearing framework in the lower mantle rocks (e.g., aggregate of bridgmanite plus ferropericlase) to control the lower mantle viscosity.

Seismic observations suggested that the substantial radial anisotropy [i.e., ξ (= (*V*_sh_/*V*_sv_)^2^)] is present beneath subduction zones due to lattice-preferred orientation of bridgmanite in dislocation creep (*17*, *32*), although the lower mantle is globally almost isotropic in terms of seismology (*32*, *33*). Global isotropy of seismic velocity indicates that diffusion creep would be dominant (*32*), although there are other possibilities (e.g., pure climb creep and complex slip systems with many equivalents). These seismic observations suggest that the deformation conditions of bridgmanite in the lower mantle were located on the boundary between the dislocation creep and the diffusion creep regimes of the deformation mechanism maps of Fig. 6. In the region where deformation is localized in the lower mantle, for example, near the subduction zones, dislocation creep would be predominant where stress is 2 × 10^4^ to 3 × 10^5^ Pa with a grain size of >3 to 8 mm as seen in Fig. 6A (dark yellow). In the central core portion of mantle convection far from the subduction zones, on the other hand, diffusion creep would dominate, where the grain size of bridgmanite is 3 to 8 mm as seen with a stress of <2 × 10^4^ to 3 × 10^5^ Pa, as shown in Fig. 6A (yellow).

One-dimensional viscosity profiles derived from geophysical observations indicate the gradual viscosity increase by one to two orders of magnitude with depths in the lower mantle, except for the lowest lower mantle. Although the pressure dependence of the viscosity of bridgmanite is not constrained in this study, the rate-limiting process of viscosity in the dislocation creep, according to activation enthalpy, would be Si and Mg self-diffusion. Bridgmanite viscosity in both dislocation creep and diffusion creep regions strongly depends on the diffusion coefficient of Si and Mg. Homologous temperature scaling (*34*) reported that the depth dependence of the diffusion coefficient for bridgmanite with adiabatic temperature gradient is consistent with the depth profiles of observed lower mantle viscosity. Therefore, the observed viscosity profiles of the whole lower mantle can be explained by that of bridgmanite with constant stress and grain size conditions. The required grain size of bridgmanite in the whole lower mantle is several millimeters from deformation mechanism map of bridgmanite. On the other hand, grain size of bridgmanite after phase transition from the transition zone with normal geotherm is estimated to be less than several hundred micrometers even by 1 billion years, because the grain growth rate of bridgmanite in a multiphase system (e.g., in lower mantle rocks) is characteristically suppressed (*35*, *36*). This indicates that the lower mantle materials located in the diffusion creep–dominant region would not have experienced the phase transition during mantle convection. Therefore, the main portion of the lower mantle would have been isolated from mixing by mantle stirring after crystallization from the magma ocean and remained as ancient mantle (*37*). Viscosity of bridgmanite in this study can help explain the presence of primordial geochemical signature such as W isotope (*38*) and noble gas isotope (*39*).

## MATERIALS AND METHODS

### Starting materials

#### Preparation of the starting materials of bridgmanite aggregates

Well-sintered MgSiO_3_ bridgmanite aggregates were prepared for starting material to calculate stress precisely, because elastic constants of Mg-endmember bridgmanite have been well determined under high pressure and high temperature conditions (*40*). At first, we synthesized enstatite aggregate following the vacuum sintering methods (*41*). Nano powders of Mg(OH)_2_ and SiO_2_ with the MgSiO_3_ stoichiometry plus H_2_O composition were well mixed by a planet type jar mill, the mixed powder was calcined in air at 1233 K for 3 hours, and the calcined powder pellets by a cold isostatic press were sintered at 1583 K for 2 hours in a vacuum condition. Complete conversion to enstatite was confirmed by the x-ray diffraction method. Bridgmanite aggregates for the deformation experiments were synthesized from the sintered enstatite aggregate at ~25 GPa and 1873 K for 20 min in the Kawai-type multianvil apparatus (6-8 cell assembly) with NaCl capsule to reduce cracks during decompression. The typical grain size of the resultant bridgmanite was several micrometers, which is suitable to obtain homogeneous Debye ring patterns in two-dimensional x-ray diffraction with a beam size of 100 to 200 μm^2^ for stress determination. Ringwoodite aggregates were synthesized at 21 GPa and 1873 K for 20 min with a similar procedure in the case of bridgmanite. Water content of ringwoodite before deformation experiments was ~40 wt parts per million (ppm) by Fourier transform infrared measurements with Paterson calibration (*42*). This amount of water in ringwoodite causes almost no effect on its creep strength (*28*). Since water solubility of Al-free bridgmanite is very low (<1 ppm) (*43*), the effect of water on deformation of bridgmanite is considered to be negligible in the present study.

### In situ stress-strain measurements during deformation experiments

The D-DIA apparatus with cubic cell assembly was previously developed on the basis of the conventional DIA-type multianvil apparatus (*44*), in which samples under high pressure can be deformed by both advancing and retracting the differential rams. This apparatus makes it possible to measure stress and strain simultaneously during deformation under high pressure conditions by adopting the in situ x-ray observation system interfaced with the synchrotron facilities. Nevertheless, the attainable pressure is still far lower than the lower mantle conditions (*28*). By using RDA (*15*), the sample can be deformed under shear geometry by rotating one anvil squeezing the sample at the lower mantle pressures; however, the deformation geometry is complicated due to combination of the shear deformation by rotation of the anvil and the uniaxial squeezing (*28*). Under these circumstances, the Kawai-type cell assembly (the 6-8 type) has been used to perform deformation experiments using both the D-DIA–type, so-called KATD, and the D111-type apparatuses (*17*, *18*, *20*, *45*). These apparatuses have enabled us to conduct deformation experiments under the lower mantle conditions.

In this study, uniaxial deformation experiments with in situ stress and strain measurements were conducted by using the D-DIA–type apparatus as KATD (*18*) at the BL04B1 beamline of the SPring-8 synchrotron facility, Hyogo, Japan and the D111-type Kawai-type apparatus (*45*) at the NE7A beamline of the Photon Factory Advanced Ring (PF-AR), National Laboratory for High Energy Accelerator Research Institute (KEK), Tsukuba, Japan. To obtain the two-dimensional x-ray diffraction patterns and x-ray radiographs, tapers and straight and conical gutters were worn on the surface of tungsten carbide (WC) second-stage cubic anvils as shown in fig. S1. Figure S2 (A and C) shows schematically the cell assemblies adopted in uniaxial deformation experiments of bridgmanite by using both the KATD and the D111 apparatuses, respectively. A cylindrical LaCrO_3_ with or without small holes for x-ray path and MgO sleeve were used as the heaters for the D111 or the KATD experiments, respectively. This difference in cell designs of x-ray window in LaCrO_3_ furnace, which also serves as thermal insulator, could produce the difference of thermal gradient through sample between the D111 apparatus and the KATD. As a result, creep strengths of bridgmanite measured by using the KATD could be slightly lower than those by using the D111 apparatus. Well-sintered hard alumina rods were used as the pistons for uniaxial deformation. Pt foils with a thickness of 10 μm were located between the sample and the piston as a strain marker. Temperature was monitored by W97%Re3%-W75%Re25% thermocouple whose junction was located next to the sample. Figure S2B shows a cell assembly for simultaneous uniaxial deformation experiments of bridgmanite and ringwoodite. Please note that bridgmanite can coexist with ringwoodite in an extremely narrow pressure range in Mg-endmember system (*46*).

White x-rays from a bending magnet were monochromatized to energies of approximately 60 keV using a Si(111) double-crystal monochrometer. Energy resolution Δ*E*/*E* is ~10^−4^ and ~10^−3^ at the BL04B1 beamline and at the NE7A beamline, respectively. X-ray radiographs of the sample were taken using an imaging system composed of a GaGG crystal and a complementary metal-oxide semiconductor camera with exposure times of 3 to 10 s as shown in Fig. 1 and fig. S3. The sample lengths were measured on x-ray radiographs. Strain at time *t*, ε(*t*),and strain rate, $\overset{\cdot }{\varepsilon }$, were calculated by using following equations: $\varepsilon (t)=-\text{ln}(\frac{h(t)}{h_{0}})$ and $\overset{\cdot }{\varepsilon }=\frac{d\varepsilon }{\mathrm{dt}}$, where *h*(*t*) is a sample thickness at time *t* and *h*_0_ is the initial sample thickness. The monochromatized x-ray was collimated to a 100 to 200 μm^2^ to obtain two-dimensional x-ray diffraction pattern of the sample using a charge-coupled device (CCD) detector and flat panel at the BL04B1 beamline and at the NE7A beamline, respectively, as shown in fig. S4. Acquisition time of x-ray diffraction pattern was 3 to 5 min. Spatial resolutions of the CCD detector and the flat panel were 99.6 and 149.6 μm, respectively. Distances from the sample to the detector at the BL04B1 beamline and at the NE7A beamline were ~720 and ~600 mm, respectively. To analyze two-dimensional diffraction patterns for the calculation of stress and pressure, “IPAnalyzer” and “PDIndexer” softwares (*47*) were used with the standard of CeO_2_ powder. Figure S5 shows converted one-dimensional x-ray diffraction patterns from fig. S4. In the D-111 apparatus, sharp MgO diffraction peaks were observed because MgO sleeve was put around the bridgmanite sample. In the KATD apparatus, although MgO including pressure medium was not put on x-ray path, broad MgO diffraction peaks from pressure medium were observed because, after compression, Boron-Epoxy in pressure medium for x-ray pass becomes thinner between second-stage anvils. To calculate stress on bridgmanite, the strongest (112) diffraction line was used with an azimuthal angle step of 6° to 20° along the ring pattern. The stress was calculated on the basis of a model showing the relationship between axial stress and lattice strain represented as the following equation (*48*)

$$d_{\text{hkl}}(\psi )=d_{\text{hkl}}^{0}[1+(1-3{\text{cos}}^{2}\psi )\frac{\sigma }{6\langle G_{\text{hkl}}\rangle }]$$ (3)

where *d*_hkl_(ψ) is the d-spacing measured as a function of azimuth angle ψ during deformation, $d_{\text{hkl}}^{0}$ is the d-spacing under the hydrostatic pressure, ⟨*G*_hkl_⟩ is Voigt-Reuss-Hill averages of the shear modulus for a given hkl, and σ is the deviatoric stress. The ⟨*G*_hkl_⟩ values were calculated using elastic constants of MgSiO_3_ bridgmanite at high pressure and temperature (*40*). The stress σ and $d_{\text{hkl}}^{0}$ were lastly determined by the least-square fitting of the observed *d*_hkl_(ψ) in Eq. 3, as shown in fig. S6A. Effect of the azimuthal angle step on stress analysis is negligibly small as shown in fig. S6B. The unit cell volumes of samples were calculated by using at least three of (111), (020), (112), and/or (200) lines from whole two-dimensional x-ray diffraction pattern, and pressures were determined on the basis of the *P-V-T* equation of state of bridgmanite (*49*), as shown in table S1.

In the experiment, the specimens were first compressed to the desired pressure at room temperature with no load on differential rams and then heated up to a target temperature and subsequently kept for 20 to 60 min to relieve stress in the specimens. At high pressure and high temperature, the differential rams were advanced at a constant speed to deform specimen. All deformation experiments in the present study were conducted under nominally dry conditions.

## Supplementary Materials

### This PDF file includes:

Figs. S1 to S9 Tables S1 and S2 References
