# Supplementary material for: Viscosity of bridgmanite determined by in situ stress and strain measurements in uniaxial deformation experiments
Source: Sci Adv. 2022 Mar 30;8(13):eabm1821. doi: 10.1126/sciadv.abm1821 (PMC8967219; doi:10.1126/sciadv.abm1821)
Supplement: Supplementary file 1 — Figs. S1 to S9 Tables S1 and S2 References [file sciadv.abm1821_sm.pdf]

Science Advances
AAAS

Supplementary Materials for

Viscosity of bridgmanite determined by in situ stress and strain measurements in uniaxial deformation experiments

Noriyoshi Tsujino*, Daisuke Yamazaki, Yu Nishihara, Takashi Yoshino, Yuji Higo, Yoshinori Tange

*Corresponding author. Email: tsujino@okayama-u.ac.jp

Published 30 March 2022, *Sci. Adv.* **8**, eabm1821 (2022)
DOI: 10.1126/sciadv.abm1821

This PDF file includes:

Figs. S1 to S9
Tables S1 and S2
References

A. KATD at SPring-8

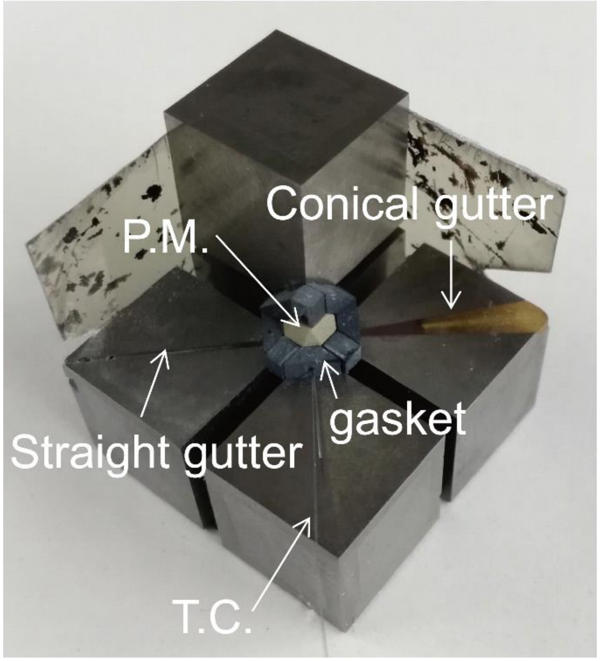

B. D111 at PF-AR, KEK

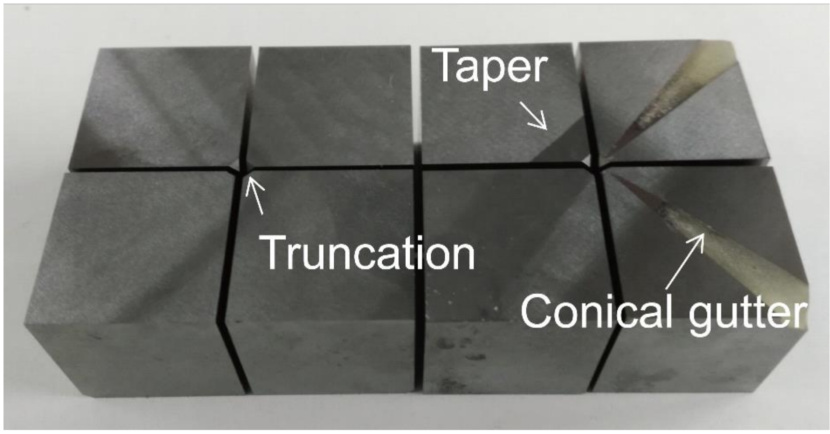

Fig. S1.

Assembly of the second stage WC anvils with conical and straight gutters or taper for (A) KATD with pressure medium and gaskets and (B) D111 apparatus.

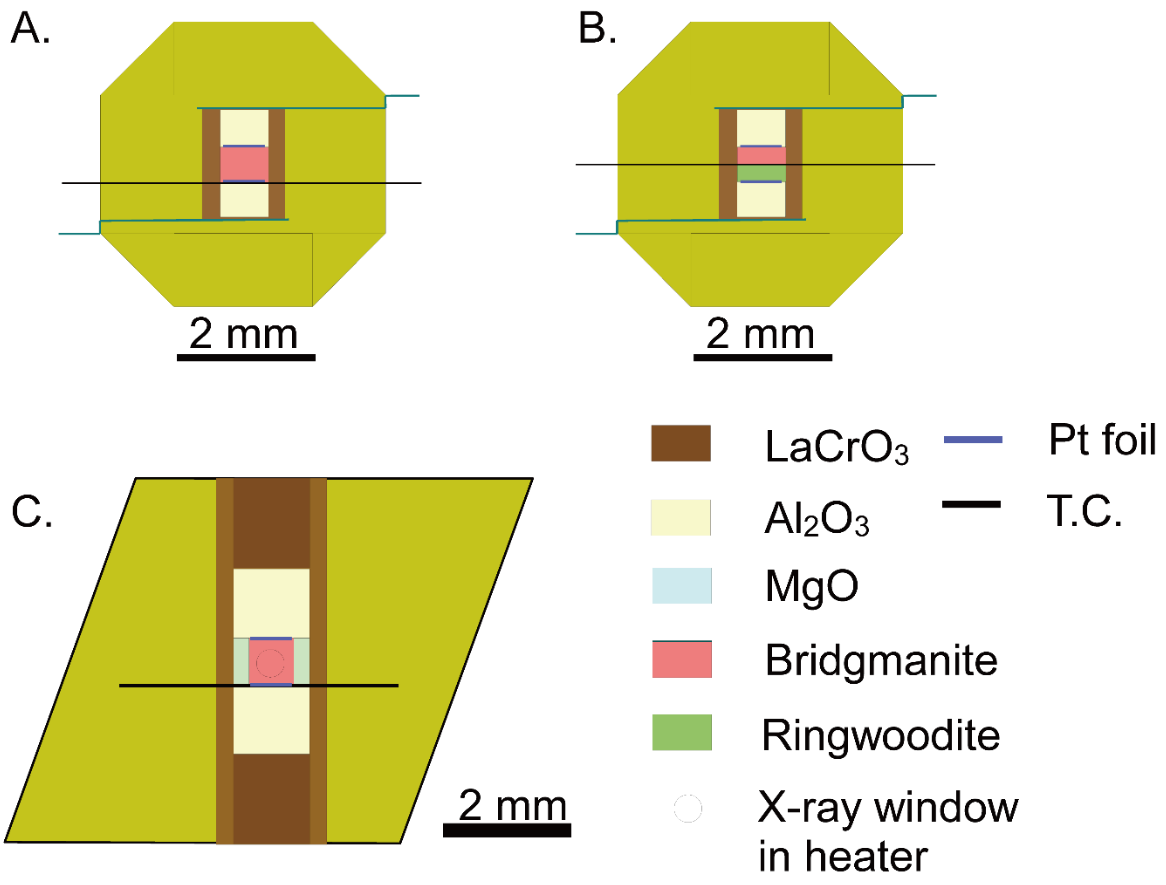

Fig. S2.

Schematic cross sections of cell assemblies for (A) uniaxial deformation experiments of bridgmanite in KATD, (B) simultaneous uniaxial deformation experiments of bridgmanite and ringwoodite in the KATD and (C) uniaxial deformation experiments of bridgmanite in the D111 apparatus with X-ray windows in the heater.

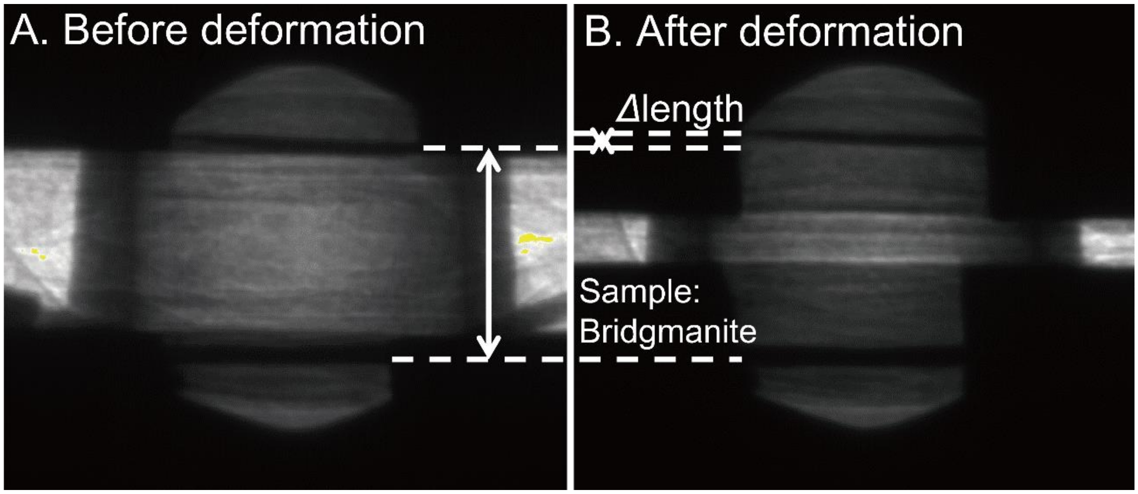

Fig. S3.

X-ray radiographs before deformation (A) and after deformation (B) in M2235 by KATD. Total strain was 4.3 % (see Table S1). *Δ*length is difference of sample length before and during deformation.

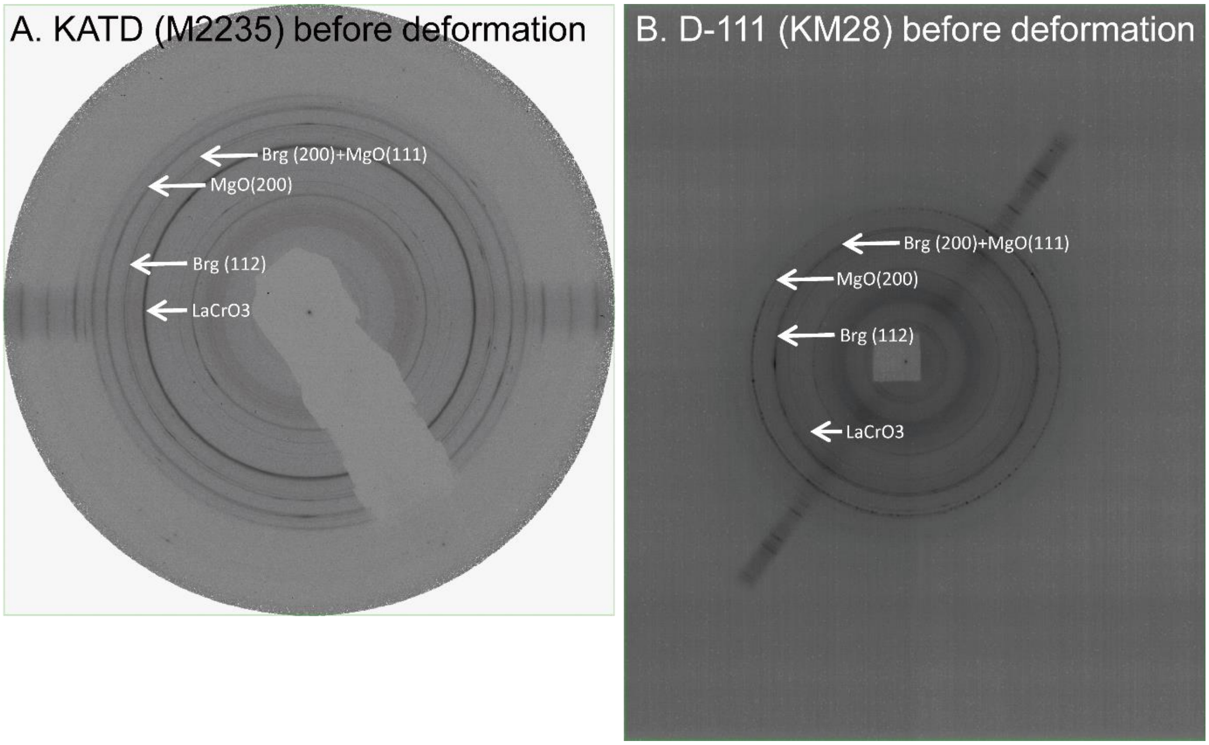

Fig. S4.

2D-monochromatic X-ray diffraction patterns at high pressure and high temperature before deformation in run M2235 in KATD at BL04B1 beamline of SPring-8 (A) and in run KM28 in D111 apparatus at NE7A beamline of PF-AR, KEK (B).

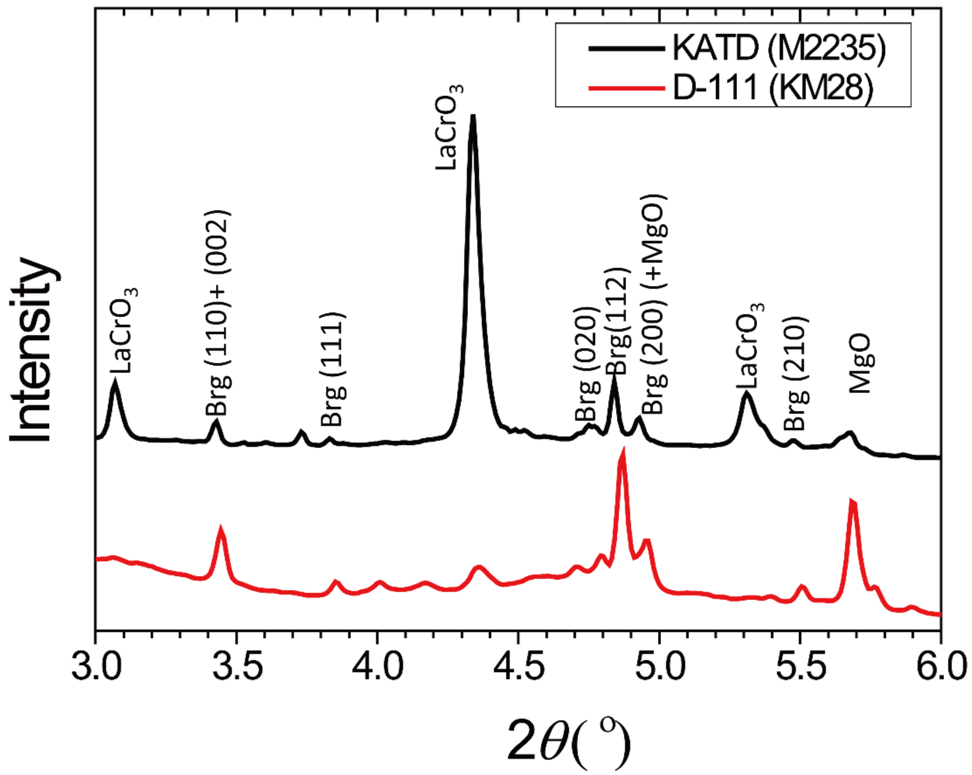

Fig. S5.

1D-monochromatic X-ray diffraction patterns at high pressure and high temperature before deformation in run M2235 in KATD at BL04B1 beamline of SPring-8 (A) and in run KM28 in D111 apparatus at NE7A beamline of PF-AR, KEK (B).

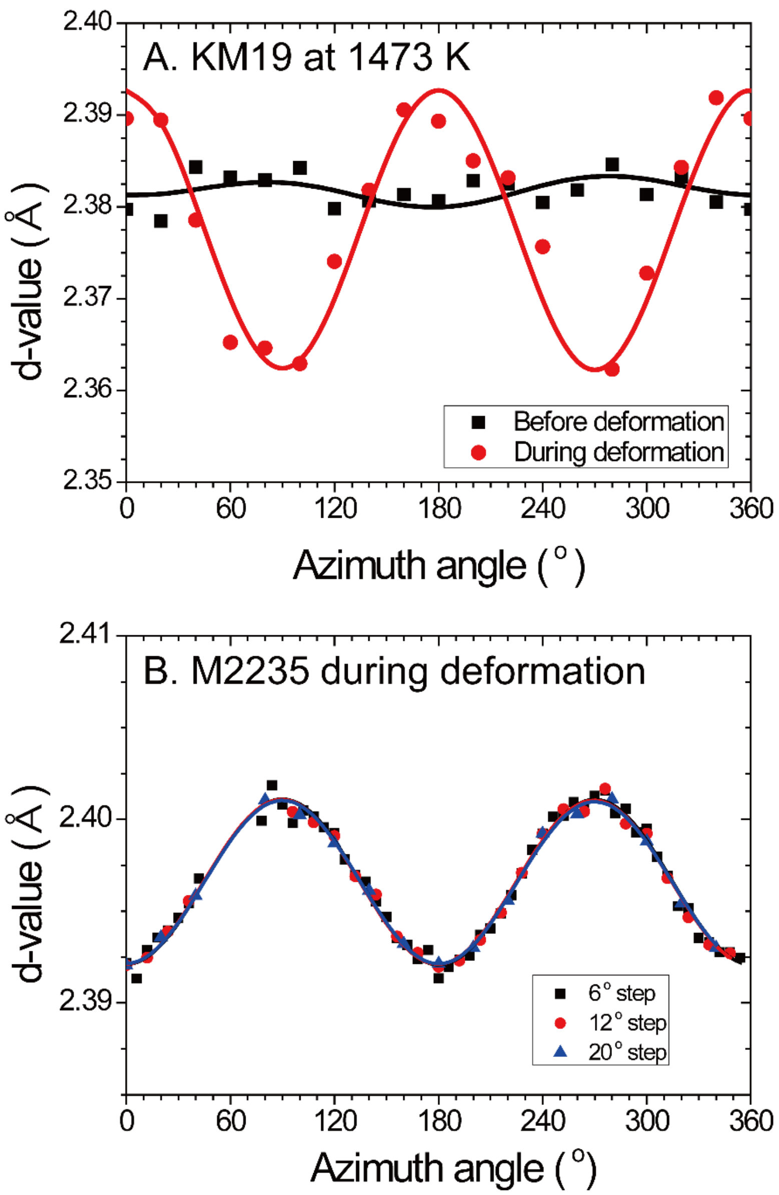

Fig. S6.

The d-spacing of bridgmanite (112) reflection vs. azimuth angle before (black) and during deformation (red) at 1473 K with displacement rate of differential ram of 10 μm/min in run KM19 (A) and during deformation with different azimuthal angle step in run M2235 (B). The 0 and 90 degrees correspond to normal and parallel directions to the uniaxial deformation direction, respectively. Black and red lines are the best fits and used to calculate stress.

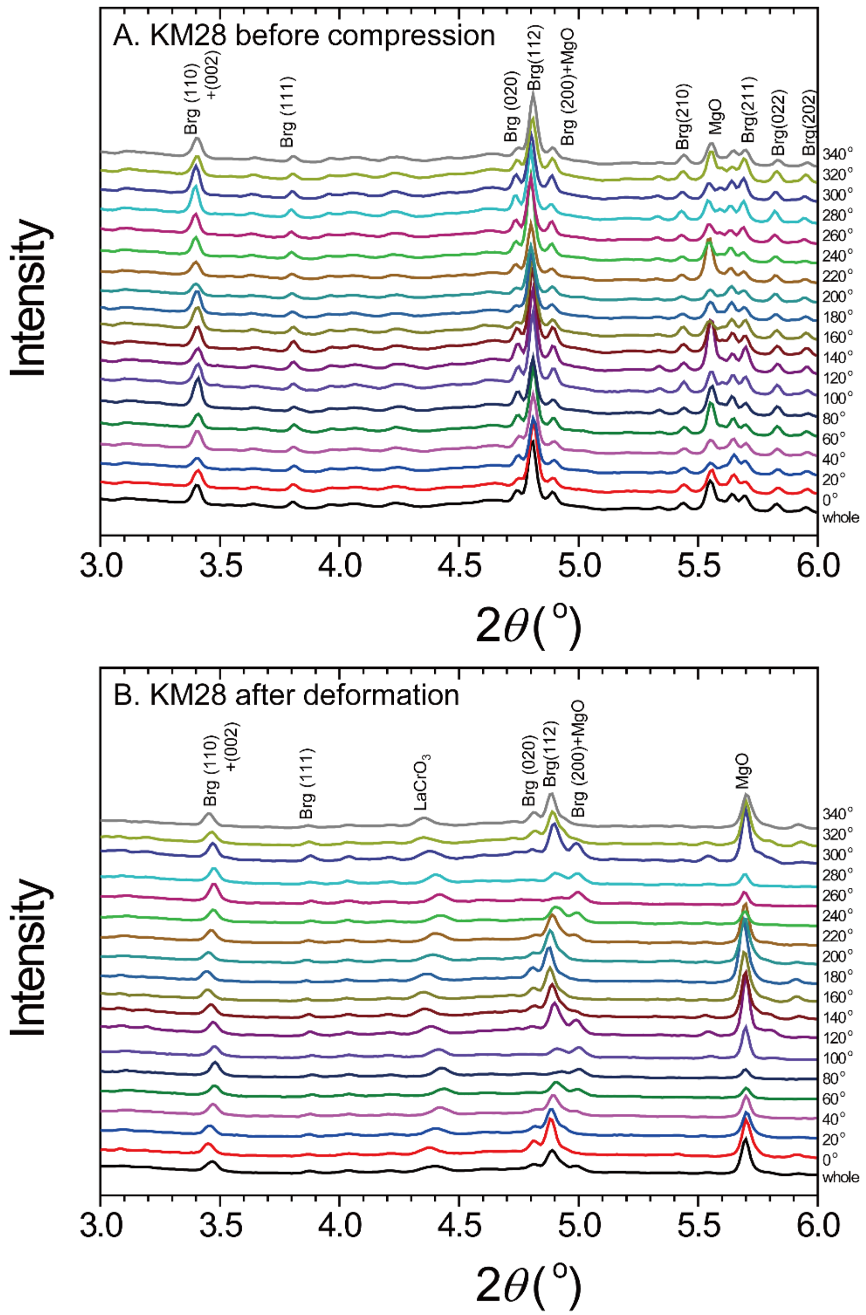

Fig. S7.
1-D x-ray diffraction with 20 ^{o} azimuthal angle step in KM28. (A) before compression and (B) after deformation. The 0 and 90 degrees correspond to normal and parallel directions to the uniaxial deformation direction, respectively. Black and red lines are the best fits and used to calculate stress. Before compression, bridgmanite peak intensity ratio against azimuthal angle is almost same while after deformation peak intensity ratio changed.

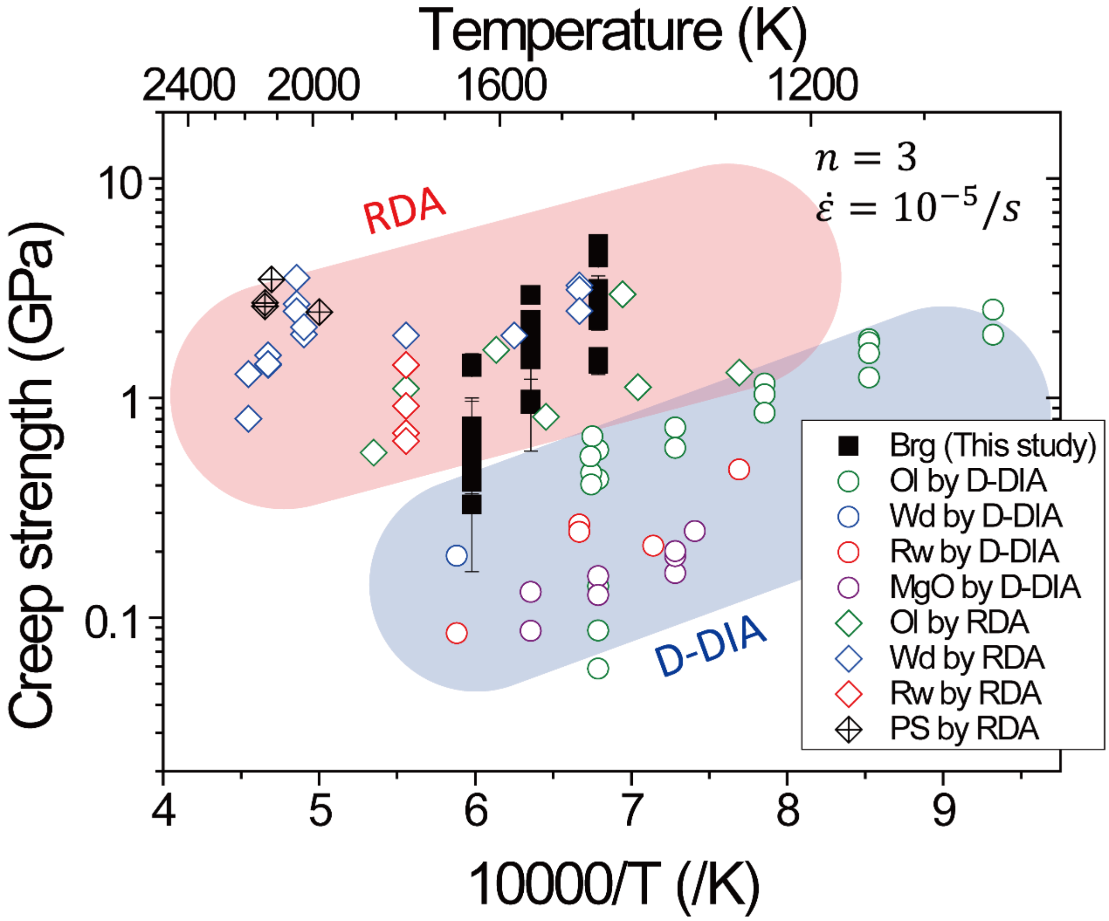

Fig. S8.

Summary of the creep strengths of dominant mantle phases with the assumption that the dominant deformation mechanism is the dislocation creep with strain-rate of 10^{-5} /s. Solid black squares represent creep strength for bridgmanite determined in this study. Open green, blue, red and purple circles represent creep strength of olivine (Ol) (25-27), wadsleyite (Wd) (28), ringwoodite (Rw) (29) and MgO (30) obtained with D-DIA apparatus, respectively. Open green, blue, red diamonds and black diamonds with cross correspond to Ol (50,51), Wd (51,52,53) Rw (52,54) and post-spinel assemble (15) obtained with RDA, respectively.

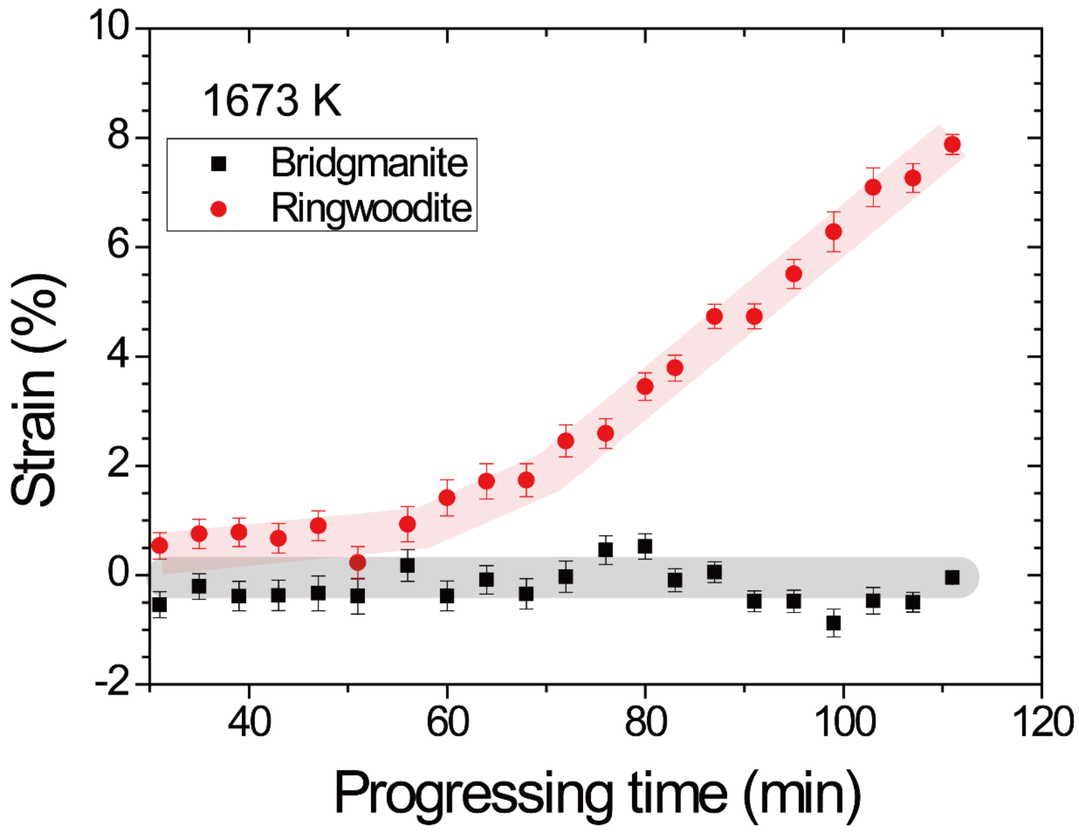

Fig. S9.

Strains of bridgmanite and ringwoodite in the simultaneous deformation experiment with progressing time at 1673 K and 23 GPa in run M2428. Total strain of ringwoodite reached ~8 % while bridgmanite displayed almost no strain. This shows that ringwoodite is substantially softer than bridgmanite.

Table S1.

Experimental conditions of bridgmanite deformation

Run No.	Temp. (K)	Pressure (GPa)	D-ram stroke speed (μm/min)	Stress (GPa)	strain rate (s^{-1})	Total strain (%)
M2309	1473	23.8-23.2	2	0.77(8)	1.6×10^{-6}	4.2
M2235	1573	23.8-22.4	2	1.12(12)	1.6×10^{-5}	4.3
M1858	1573	24-7-23.1	2	0.59(24)	2.2×10^{-6}	2.6
M2344	1573	23.7-22.4	2	0.57(3)	2.4×10^{-6}	6.8
M1862	1673	22.8-22.3	1	0.25(12)	4.5×10^{-6}	2.2
M1854	1673	23.1-22.8	2	0.44(12)	1.3×10^{-5}	5.5
M1866	1673	23.7-23.5	3	0.73(14)	2.1×10^{-5}	4.3
KM19	1473	27.0-23.9	1	2.6(4)	6.1×10^{-6}	21.0
	1473		10	4.5(5)	3.5×10^{-5}	
	1573		3	2.3(4)	1.8×10^{-5}	
	1673		3	0.9(4)	2.5×10^{-5}	
KM28	1473	26.5-22.6	3	2.7(3)	2.0×10^{-5}	27.7
	1473		1	2.0(2)	6.6×10^{-6}	
	1473		3	3.0(3)	1.7×10^{-5}	
KM30	1573	26.8-23.6	1	1.3(1)	2.9×10^{-6}	24.3
	1573		3	1.9(2)	1.9×10^{-5}	
	1573		10	2.9(2)	4.8×10^{-5}	
KM24	1573	27.8-23.1	3	2.0(4)	2.7×10^{-5}	30.1
	1673		3	1.1(4)	3.6×10^{-5}	
	1673		10	1.1(3)	1.5×10^{-4}	
KM32	1673	26.7-25.5	1	1.4(2)	1.2×10^{-5}	28.0
	1573		1	2.4(2)	7.0×10^{-6}	
	1473		1	3.6(3)	4.9×10^{-6}	
KM34	1673	27.6-23.6	3	1.7(1)	1.9×10^{-5}	14.2
	1573		3	2.5(2)	1.4×10^{-5}	
	1573		3	2.3(2)	1.5×10^{-5}	
	1473		3	3.9(2)	7.6×10^{-6}	

M series of Run No. were conducted by using KATD, whereas KM series of Run No. were conducted by using D111 apparatus.

Table S2.

Best fit parameters in dislocation creep regime

	log A ($GPa^{-n}s^{-1}$)	H (kJ/mol)
	n = 3 (fix)	
KM19	9.7(26)	456(77)
KM32	6.5(15)	382(46)
KM34	5.6(12)	350(35)
All data by D111	7.1(20) *	385(59) *
All data by KATD	8.7(25)	405(77)
	n = 3.5 (fix)	
All data by D111	8.5(21) *	432(64) *
All data by KATD	9.9(31)	440(98)

*: These values are used as parameters to construct the deformation mechanism maps of bridgmanite at 25 GPa and 1900 K with lattice diffusion coefficient D_l of 1.6×10^{-19} m^2/s and grain boundary diffusion coefficient δD_{gb} of 2.0×10^{-25} m^3/s (5).

REFERENCES AND NOTES

1. Y. Ricard, B. Wuming, Inferring the viscosity and the 3-D density structure of the mantle from geoid, topography and plate velocities. *Geophys. J. Int.* **105**, 561–571 (1991).

2. A. M. Forte, J. X. Mitrovica, New inferences of mantle viscosity from joint inversion of long-wavelength mantle convection and post-glacial rebound data. *Geophys. Res. Lett.* **23**, 1147–1503 (1996).

3. B. M. Steinberger, A. R. Calderwood, Mineral physics constraints on viscous models of mantle. *J. Conf. Abs.* **6**, (2001).

4. J. X. Mitrovica, A. M. Forte, A new inference of mantle viscosity based upon joint inversion of convection and glacial isostatic adjustment data. *Earth Planet. Sci. Lett.* **225**, 177–189 (2004).

5. D. Yamazaki, T. Kato, H. Yurimoto, E. Ohtani, M. Toriumi, Silicon self-diffusion in $MgSiO_3$ perovskite at 25 GPa. *Phys. Earth Planet. Inter.* **119**, 299–309 (2000).

6. D. P. Dobson, R. Dohmen, M. Wiedenbeck, Self-diffusion of oxygen and silicon in $MgSiO_3$ perovskite. *Earth Planet. Sci. Lett.* **270**, 125–129 (2008).

7. J. Xu, D. Yamazaki, T. Katsura, X. Wu, P. Remmert, H. Yurimoto, S. J. Chakraborty, Silicon and magnesium diffusion in a single crystal of $MgSiO_3$ perovskite. *J. Geophys. Res.*, **116**, B12205 (2011).

8. H. Fei, D. Yamazaki, M. Sakurai, N. Miyajima, H. Ohfuji, T. Katsura, T. Yamamoto, A nearly water-saturated mantle transition zone inferred from mineral viscosity. *Sci. Adv.* **3**, e1603024 (2017).

9. T. Yoshino, G. Manthilake, T. Matsuzaki, T. Katsura, Dry mantle transition zone inferred from the conductivity of wadsleyite and ringwoodite. *Nature* **451**, 326–329 (2008).

10. X. Huang, Y. Xu, S. Karato, Water content in the transition zone from electrical conductivity of wadsleyite and ringwoodite. *Nature* **434**, 746–749 (2005).

11. A. Kelbert, A. Schultz, G. Egbert, Global electromagnetic induction constraints on transition-zone water content variations. *Nature* **460**, 1003–1006, (2009).

12. S. Merkel, H.-R. Wenk, J. Badro, G. Montagnac, P. Gilliet, H. Mao, R. J. Hemley, Deformation of $(Mg_{0.9},Fe_{0.1})SiO_3$ Perovskite aggregates up to 32 GPa. *Earth Planet. Sci. Lett.* **209**, 351–360 (2003).

13. L. Miyagi, H. R. Wenk, Texture development and slip systems in bridgmanite and bridgmanite + ferropericlase aggregates. *Phys. Chem. Minerals*, **43**(8), 597–613 (2016).

14. J. Chen, D. J. Weidner, M. T. Vaughan, The strength of $Mg_{0.9}Fe_{0.1}SiO_3$ perovskite at high pressure and temperature. *Nature* **419**, 824–826 (2002).

15. J. Girard, G. Amulele, R. Farla, A. Mohiuddin, S. Karato, Shear deformation of bridgmanite and magnesiowüsitite aggregates at lower mantle conditions. *Science* **351**, 144–147, (2016).

16. F. Boioli, P. Carrez, P. Cordier, B. Devincre, K. Gouriet, P. Hirel, A. Kraych, S. Rittrebex, Pure climb creep mechanism drives flow in Earth's lower mantle. *Sci. Adv.* **3**, e1601958 (2017).

17. N. Tsujino, Y. Nishihara, D. Yamazaki, Y. Seto, Y. Higo, E. Takahashi, Mantle dynamics inferred from the crystallographic preferred orientation of bridgmanite. *Nature* **539**, 81–84 (2016).

18. N. Tsujino, Rheological study of bridgmanite at the lower mantle. *Rev. High Press. Sci. Tech.* **28**, 139–148 (2018).

19. S. Couper, S. Speziale, H. Mrarquardt, H. P. Liermann, L. Miyagi, Does heterogenous strain act as a control on seismic anisotropy in Earth's lower mantle? *Front. Earth Sci.* **8**, 540449 (2020).

20. S. A. Hunt, D. J. Weidner, R. J. McCormack, M. L. Whitaker, E. Bailey, L. Li, M. T. Vaughan, D. P. Dobson, Deformation T-Cup: A new multi-anvil apparatus for controlled

strain-rate deformation experiments at pressures above 18 GPa. *Rev. Sci. Instrum.* **85**, 085103 (2014).

21. G. Hirth, D. Kohlstedt, Rheology of the upper mantle and the mantle wedge: A view from the experimentalists, in *Inside the Subduction Factory*, J. Eiler, Ed. (AGU, 2003), pp. 83–105.

22. J. Korenaga, S. Karato, A new analysis of experimental data on olivine rheology. *J. Geophys. Res.* **113**, B02403 (2008).

23. H. J. Frost, M. F. Ashby, *Deformation Mechanisms Maps* (Pergamon press, Oxford, 1982).

24. L. Li, D. Weidner, P. Raterron, J. Chen, M. Vaughan, S. Mei, B. Durhan, Deformation of olivine at mantle pressure using the D-DIA. *Eur. J. Mineral.* **18**, 7–19 (2006).

25. S. Kaboli, O. C. Burnley, G. Xia, H. W. Green II, Pressure dependence of creep in forsterite olivine: Comparison of measurements from the D-DIA and Griggs apparatus. *Geophys. Res. Lett.* **44**, 10939–10947 (2017).

26. H. Long, D. J. Weidner, L. Li, J. Chen, L. Wang, Deformation of olivine at subduction zone conditions determined from in situ measurements with synchrotron radiation. *Phys. Earth Plant. Inter.* **186**, 23–35 (2011).

27. T. Kawazoe, Y. Nishihara, T. Ohuchi, N. Nishiyama, Y. Higo, K. Funakoshi, T. Irifune, In situ stress-strain measurements in a deformation-DIA apparatus at P-T conditions of the upper part of the mantle transition zone. *Am. Mineral.* **96**, 1665–1672 (2011).

28. T. Kawazoe, Y. Nishihara, T. Ohuchi, N. Miyajima, G. Maruyama, Y. Higo, K. Funakoshi, T. Irifune, Creep strength of ringwoodite measured at pressure–temperature conditions of the lower part of the mantle transition zone using a deformation–DIA apparatus. *Earth Planet. Sci. Lett.* **454,** 10–19 (2016).

29. S. Mei, D. L. Kohlstedt, W. B. Durham, L. Wang, Experimental investigation of the creep behavior of MgO at high pressures. *Phys. Earth Planet. Inter.* **170**, 170–175 (2008).

30. X. Chu, J. Korenaga, Olivine rheology, shear stress, and grain growth in the lithospheric mantle: Geological constraints from the Kaapvaal craton. *Earth Planet. Sci. Lett.* **333-334**, 52–62, (2012).

31. D. L. Kohlstedt, L. N. Hansen, Constitutive Equations, Rheological Behavior, and Viscosity of Rocks, in *Treatise on Geophysics* (Elsevier, 2015), pp. 441–472, doi.org/10.1016/B978-0-444-53802-4.00042-7

32. A. M. G. Ferreira, M. Faccenda, W. Sturgeon, S. Chang, L. Schardong, Ubiquitous lower-mantle anisotropy beneath subduction zones. *Nat. Geosci.* **12**, 301–306, (2019).

33. M. Panning, B. Romanowicz, A three-dimensional radially anisotropic model of shear velocity in the whole mantle. *Geophys. J. Int.* **167,** 361–379 (2006).

34. D. Yamazaki, S. Karato, Some mineral physics constraints on the rheology and geothermal structure of Earth's lower mantle. *Am. Mineral.* **86**:385–391 (2001).

35. D. Yamazaki, T. Kato, E. Ohtani, M. Toriumi, Grain growth rates of $MgSiO_3$ perovskite and periclase under lower mantle conditions. *Science* **274**:2052–2054 (1996).

36. M. Imamura, Experimental study on rheological properties of mantle minerals: Implication for subducting slab and the lower mantle, thesis, Kyushu University (2018).

37. M. D. Ballmer, C. Houser, J. W. Hernlund, R. Wentzcovich, K. Hirose, Persistence of strong silica-enriched domains in the Earth's lower mantle. *Nat. Geosci.* **10**, 236–240 (2017).

38. H. Rizo, R. J. Walker, R. W. Carlson, M. F. Horan, S. Mukhopadhyay, V. Manthos, D. Francis, M. G. Jackson, Preservation of Earth-forming events in the tungsten isotopic composition of modern flood basalts. *Science* **352**, 809–812 (2016).

39. S. Mukhopadhyay, Early differentiation and volatile accretion recorded in deep-mantle neon and xenon. *Nature* **486**, 101–104 (2012).

40. R. M. Wentzcovitch, B. B. Karki, M. Cococcioni, S. de Gironcoli, Thermoelastic properties of MgSiO3-perovskite: Insights on the nature of the earth's lower mantle. *Phys. Rev. Lett.* **92**, 018501 (2004).

41. S. Koizumi, T. Hiraga, C. Tachibana, M. Tasaka, T. Miyazaki, T. Kobayashi, A. Takamasa, N. Ohashi, S. Sano, Synthesis of highly dense and fine-grained aggregates of mantle composites by vacuum sintering of nano-sized mineral powders. *Phys. Chem. Miner.* **37**, 505–518 (2010).

42. M. S. Paterson, The determination of hydroxyl by infrared absorption in quartz, silicate glasses and similar materials. *Bull. Miner.* **105**, 20–29. (1982).

43. N. Bolfan-Casanova, H. Keppler, D. C. Rubie, Water partitioning between nominally anhydrous minerals in the MgO–SiO2–H2O system up to 24 GPa: Implications for the distribution of water in the Earth's mantle. *Earth Planet. Sci. Lett.* **182**, 209–221 (2000).

44. Y. Wang, W. Durham, I. C. Getting, D. J. Weidner, The deformation-DIA: A new apparatus for high temperature triaxial deformation to pressures up to 15 GPa. *Rev. Sci. Instrum.* **74**, 3002 (2003).

45. Y. Nishihara, N. Tsujino, T. Kubo, D. Yamazaki, S. Doi, M. Imamura, T. Yoshino, Studies of deep Earth rheology based on high-pressure deformation experiments using D111-type apparatus. *Rev. High Press. Sci. Tech.* **30**, 78–84, (2020).

46. T. Ishii, H. Kojitani, M. Akaogi, Post-spinel transitions in pyrolite and Mg_2SiO_4 and akimotoite–perovskite transition in $MgSiO_3$: Precise comparison by high-pressure high-temperature experiments with multi-sample cell technique. *Earth Planet. Sci. Lett.* **309,** 185–197 (2011).

47. Y. Seto, D. Nishio-Hamane, T. Nagai, N. Sata, Development of a software suite on x-ray diffraction experiments. *Rev. High Press. Technol.* **20**, 269–276 (2010).

48. A. K. Singh, C. Balasingh, H. K. Mao, R. Hemley, J. Shu, Analysis of lattice strains measured under nonhydrostatic pressure. *J. Appl. Phys.* **83**, 7567–7575. (1998).

49. Y. Tange, Y. Kuwayama, T. Irifune, K. Funakoshi, Y. Ohishi, *P-V-T* equation of state of $MgSiO_3$ perovskite based on the MgO pressure scale: A comprehensive reference for mineralogy of the lower mantle. *J. Geophys. Res.* **117**, B06201 (2012).

50. Y. Nishihara, D. Tinker, T. Kawazoe, Y. Xu, Z. Jing, K. N. Matsukage, S. Karato, Plastic deformation of wadsleyite and olivine at high-pressure and high-temperature using a rotational Drickamer apparatus (RDA). *Phys. Earth Planet. Inter.* **170**, 156–169 (2008).

51. T. Kawazoe, S. Karato, K. Otsuka, Z. C. Jing, M. Mookherjee, Shear deformation of dry polycrystalline olivine under deep upper mantle conditions using a rotational Drickamer apparatus (RDA). *Phys. Earth Planet. Inter.* **174**, 128–137, (2009).

52. J. Hustoft, G. Amulele, J. Ando, K. Otsuka, Z. X. Du, Z. C. Jing, S. Karato, Plastic deformation experiments to high strain on mantle transition zone minerals wadsleyite and ringwoodite in the rotational Drickamer apparatus. *Earth Planet. Sci. Lett.* **361**, 7–15 (2013).

53. R. Farla, G. Amulele, J. Girard, N. Miyajima, S. Karato, High-pressure and high-temperature deformation experiments on polycrystalline wadsleyite using the rotational Drickamer apparatus. *Phys. Chem. Minerals* **42**, 541–558 (2015).

54. L. Miyagi, G. Amulele, K. Otsuka, Z. X. Du, R. Farla, S. Karato, Plastic anisotropy and slip systems in ringwoodite deformed to high shear strain in the rotational Drickamer apparatus. *Phys. Earth Planet. Inter.* **228,** 244–253 (2014).
